# A rapid and effective method for screening, sequencing and reporter verification of engineered frameshift mutations in zebrafish

**DOI:** 10.1242/dmm.026765

**Published:** 2017-06-01

**Authors:** Sergey V. Prykhozhij, Shelby L. Steele, Babak Razaghi, Jason N. Berman

**Affiliations:** 1 Department of Pediatrics, Dalhousie University, Halifax, NS, Canada B3K 6R8; 2 Department of Microbiology and Immunology, Dalhousie University, Halifax, NS, Canada B3H 4R2; 3 Department of Pathology, Dalhousie University, Halifax, NS, Canada B3H4R2

**Keywords:** CRISPR, Cas9, SgRNA, Zebrafish, Mutation, Reporter

## Abstract

Clustered regularly interspaced palindromic repeats (CRISPR)/Cas-based adaptive immunity against pathogens in bacteria has been adapted for genome editing and applied in zebrafish (*Danio rerio*) to generate frameshift mutations in protein-coding genes. Although there are methods to detect, quantify and sequence CRISPR/Cas9-induced mutations, identifying mutations in F1 heterozygous fish remains challenging. Additionally, sequencing a mutation and assuming that it causes a frameshift does not prove causality because of possible alternative translation start sites and potential effects of mutations on splicing. This problem is compounded by the relatively few antibodies available for zebrafish proteins, limiting validation at the protein level. To address these issues, we developed a detailed protocol to screen F1 mutation carriers, and clone and sequence identified mutations. In order to verify that mutations actually cause frameshifts, we created a fluorescent reporter system that can detect frameshift efficiency based on the cloning of wild-type and mutant cDNA fragments and their expression levels. As proof of principle, we applied this strategy to three CRISPR/Cas9-induced mutations in *pycr1a*, *chd7* and *hace1* genes. An insertion of seven nucleotides in *pycr1a* resulted in the first reported observation of exon skipping by CRISPR/Cas9-induced mutations in zebrafish. However, of these three mutant genes, the fluorescent reporter revealed effective frameshifting exclusively in the case of a two-nucleotide deletion in *chd7*, suggesting activity of alternative translation sites in the other two mutants even though *pycr1a* exon-skipping deletion is likely to be deleterious. This article provides a protocol for characterizing frameshift mutations in zebrafish, and highlights the importance of checking mutations at the mRNA level and verifying their effects on translation by fluorescent reporters when antibody detection of protein loss is not possible.

## Abstract (precis)

**Summary:** A new CRISPR/Cas9-based method of fluorescent reporter technology in zebrafish to confirm loss of gene function in this model.

## INTRODUCTION

The clustered regularly interspaced palindromic repeats (CRISPR)/Cas9 system currently in use for genome editing is derived from the type II CRISPR/Cas system in several bacterial species, where it serves as adaptive immunity against molecular pathogens by inducing cleavage of their DNA (Sorek et al., 2013). The original demonstrations that CRISPR RNA with tracrRNA or engineered single-guide RNA (sgRNA) created from CRISPR RNA and tracrRNA can program Cas9 nuclease specificity *in vitro* (Gasiunas et al., 2012; Jinek et al., 2012) and in cells (Cho et al., 2013; Cong et al., 2013; Mali et al., 2013) have opened the floodgates for work on genome editing, one application of which is to generate inactivating mutations in protein-coding genes either by targeting single sgRNA sites to create frameshifts or inducing larger gene deletions with multiple sgRNAs. When introduced into cells, Cas9/sgRNA complexes induce double-strand breaks at defined sequences, which are typically repaired by the error-prone non-homologous end-joining or microhomology-mediated end-joining DNA repair pathways resulting in small insertions or deletions (indels) (Rodgers and Mcvey, 2016).

Most indels in protein-coding gene exons are predicted to be frameshift mutations that disrupt open reading frames with the obvious exception of those for which the size is a multiple of three. Frameshift mutations are potentially highly suitable for generating loss-of-function mutations in protein-coding genes. However, there are several challenges to overcome in order for this type of mutation to become an easy and reliable tool for gene characterization. The first challenge is related to target site selection because targeting too close to the natural translational initiation codon might be suboptimal owing to the fact that cells could utilize alternative start codons in many of their genes. By contrast, targeting downstream exons might generate a somewhat functional hypomorphic mutant version of the gene. The mechanism of translation start site selection is currently described by the scanning model: 40S ribosomal subunits bind to the 5′ cap with the help of initiation factors and then scan mRNA in a 5′ to 3′ direction for the first suitable translation start site located in an appropriate sequence context whereupon 60S ribosomal subunit is recruited and translation begins (Hinnebusch, 2011). Alternative translation start sites can be used as a result of leaky scanning when the ribosome scans past the first initiation site and initiates translation at one of the downstream sites or when re-initiation happens after a short peptide (10-30 amino acids) has been translated and termination has occurred (Kochetov, 2008). Utilization of alternative start codons depends on their sequence context and the overall structure of mRNA. There are many known examples of alternative start sites due to leaky scanning being used for generating protein isoforms differing by localization, as reviewed by Kochetov (2008). Recent evidence suggests the presence of upstream open reading frames (uORFs) in approximately half of mammalian mRNAs (Ingolia et al., 2011), which means that translation of main ORFs in these mRNAs requires translation re-initiation. uORFs typically reduce translation efficiency or can completely block translation of the main ORF under certain conditions, but when the main ORF in mRNAs containing uORFs starts translation, its initiation can involve either the leaky scanning mechanism or translation re-initiation (Barbosa et al., 2013). Frameshift mutations close to the normal translation start site will lead to early translation termination, which will either cause nonsense-mediated decay or will make the resulting short ORFs behave akin to uORFs with the potential to block downstream translation or result in translation re-initiation leading to truncated protein products. Moreover, frameshift mutations in the main ORF are not very likely to impact uORF-mediated regulation, but rather might further inhibit translation of genes under such uORF-mediated regulation. On the other hand, the leaky-scanning mechanism implies that the usage of alternative start codons downstream of frameshift mutations can bypass their negative effects resulting in a truncated protein. These findings related to alternative translation start sites mean that some CRISPR/Cas9-generated mutations predicted to be frameshift mutations will turn out to be ineffective because they are located upstream of an effective alternative translation start site.

In contrast to mutant generation using forward genetics methods in which one starts with phenotypes, mutants generated by CRISPR/Cas9 or other genome editing techniques are designed in particular genes but there is no guarantee of a phenotype especially if the mutations fail to inactivate the genes. Generating zebrafish mutants using genome editing is certainly easier than with forward genetics approaches but there are a few challenges to overcome. One of the problems for mutant description is a paucity of affordable available antibody reagents for zebrafish proteins. This challenge leads to frequent reliance on simple logical inferences from gel analysis and sequencing data that certain mutations will abolish the standard reading frame and therefore must be null. Such inferences are valid when there is phenotypic support, but they can fail when there is no such data and there is still a question of whether the protein is non-essential or whether the effect of the mutation is not significant, for example due to alternative translation start sites or alterations to splicing. Generation of deletion mutants using CRISPR/Cas9 or transcription activator-like effector nucleases (TALENs) represents a valid strategy for addressing this issue, but developing such strategies is more complex, the frequency of successful deletions can be low and might not be fully applicable outside of the context of generating single-gene mutations, such as when genetic screens are performed using sgRNA pools or when multiple sgRNAs are expressed transgenically. In these multiplexed systems it is desirable to have a few sgRNAs per gene.

In zebrafish, CRISPR/Cas9 technology has been most often used to introduce frameshift mutations in particular genes (for examples, please see Gagnon et al., 2014; Varshney et al., 2015a). Initial detection of mutation induction by sgRNA/Cas9 complexes most often relies on commercially available T7 Endonuclease I or SURVEYOR (Cel-I) nuclease, which can detect bulges in heteroduplex PCR products containing indels and cut them into two pieces. These approaches are adequate for small numbers of samples but become too laborious for screening larger numbers of zebrafish. Another method most suitable for characterizing sgRNA effectiveness in initial injections is CRISPR-STAT, which involves fluorescent PCR followed by capillary gel electrophoresis of PCR products (Carrington et al., 2015). Screening large numbers of fish, for example F1 mutation carriers, F2 heterozygotes or many embryos/larvae in F1 or subsequent generations, requires methods with significantly higher throughput. Several studies in zebrafish developed high-resolution melting analysis (HRMA), a method similar to quantitative PCR (qPCR) but focused mainly on detecting changes in melting curves of the amplicons due to mutations (Dahlem et al., 2012; Talbot and Amacher, 2014; Thomas et al., 2014). HRMA offers simple sample preparation and high throughput but typically requires specialized equipment and/or software, though a recent study suggests that standard qPCR equipment can be optimized for HRMA to make it more broadly accessible (D'Agostino et al., 2015). An even easier genotyping method to implement is the heteroduplex mobility assay (HMA) because it relies on the slower mobility of heteroduplex PCR products containing mutations in the polyacrylamide gel electrophoresis (PAGE) (Chen et al., 2012; Ota et al., 2013, 2014). HMA is straightforward to perform without much specialized equipment, a feature attractive to many labs starting to work with CRISPR/Cas9. The throughput of HMA is lower than that of HRMA (Dahlem et al., 2012; Talbot and Amacher, 2014; Thomas et al., 2014) or fluorescent PCR approaches of the CRISPR-STAT (Carrington et al., 2015) but HMA still allows analysis of 20-40 samples in a single run with the time depending on the features of the PAGE apparatus and amplicon size.

Based on the need to screen and verify which CRISPR/Cas9 mutations are effective at shifting the translation reading frame in protein-coding genes, we developed a reporter assay for measuring frameshift effectiveness. The underlying question for this method is to what extent indel mutations predicted to terminate translation prematurely do so in reality. This question pertains very frequently to interpretation of mutations in many model systems in which CRISPR/Cas9 is applied and new approaches could be helpful in addressing it. We have focused on zebrafish because we work with this model species, but the method can, in principle, be applied to many other model species, in which CRISPR/Cas9 and mRNA injections as well as genotyping of adult animals are possible. Another important feature of this method is that it can ideally be applied to F1 animals to shorten the waiting period for mutant characterization and establishment of F2 mutation carrier lines for the most effective mutations. We use HMA to genotype F1 mutation carriers as well as bacterial clones derived by cloning amplified fragments of the targeted genome sites. To improve throughput and to identify clones corresponding to individual F1 zebrafish unambiguously, we apply barcoding and multiplexing strategies to identify the precise nature of the mutations. The mutation reporter strategy we use to assess the frameshift potential of mutations relies on cloning cDNA fragments containing 5′ UTRs as well as the first 200 codons from wild-type and mutant cDNAs in-frame with sfGFP fluorescent protein gene and separated by the P2A element for co-translational protein cleavage. Application of this strategy to several zebrafish mutations confirmed its effectiveness and also resulted in identification of a mutation causing an exon-skipping event, for which we proposed a molecular mechanism based on bioinformatics predictions. Overall, this resource article should be useful for screening and deeper characterization of mutants in many model species.

## RESULTS

### Screening of F1 mutation carriers and identifying their mutations

Given zebrafish generation time of about 3 months, it takes 3-4 months to obtain adult founder (F0) animals, some of which carry from one to several mutations distributed in a mosaic fashion, which makes them difficult to use for consistent mutational analysis experiments. The next generation (F1) produced by outcrossing founders to wild-type zebrafish of the same strain will carry either no mutation or a single mutation per fish. It is thus possible to obtain multiple F1 adult zebrafish with deleterious mutations and breed them to produce compound heterozygote or even homozygote mutant embryos and determine whether these embryos/larvae have a phenotype due to loss of the targeted gene. This approach can save a generation time for an average project and can also help prioritize which mutations should be used for establishing a mutant line. We use HMA as a method to detect the presence of indels in a heterozygous genetic background. HMA relies on the much slower mobility of heteroduplex DNA molecules in a polyacrylamide gel and is influenced by the length of the indel, its distance from the center of the PCR product and the percentage of the polyacrylamide gel used. The advantages of HMA are that no purification of the PCR reactions or enzymatic digestions is required for mutation detection. HMA protocols have been presented for genotyping zebrafish injected with zinc-finger nucleases (Chen et al., 2012), TALENs (Ota et al., 2013) and CRISPR/Cas9 (Ota et al., 2014) reagents, but these papers did not describe their HMA procedures in sufficient detail or focused on mutation detection in very small PCR products. We present a more generalized and detailed HMA version for zebrafish genotyping (see supplementary information), which can be adapted to any PCR products in the 100-600 bp size range and integrate it into our workflow for characterizing engineered mutations in zebrafish.

**Fig. 1.**
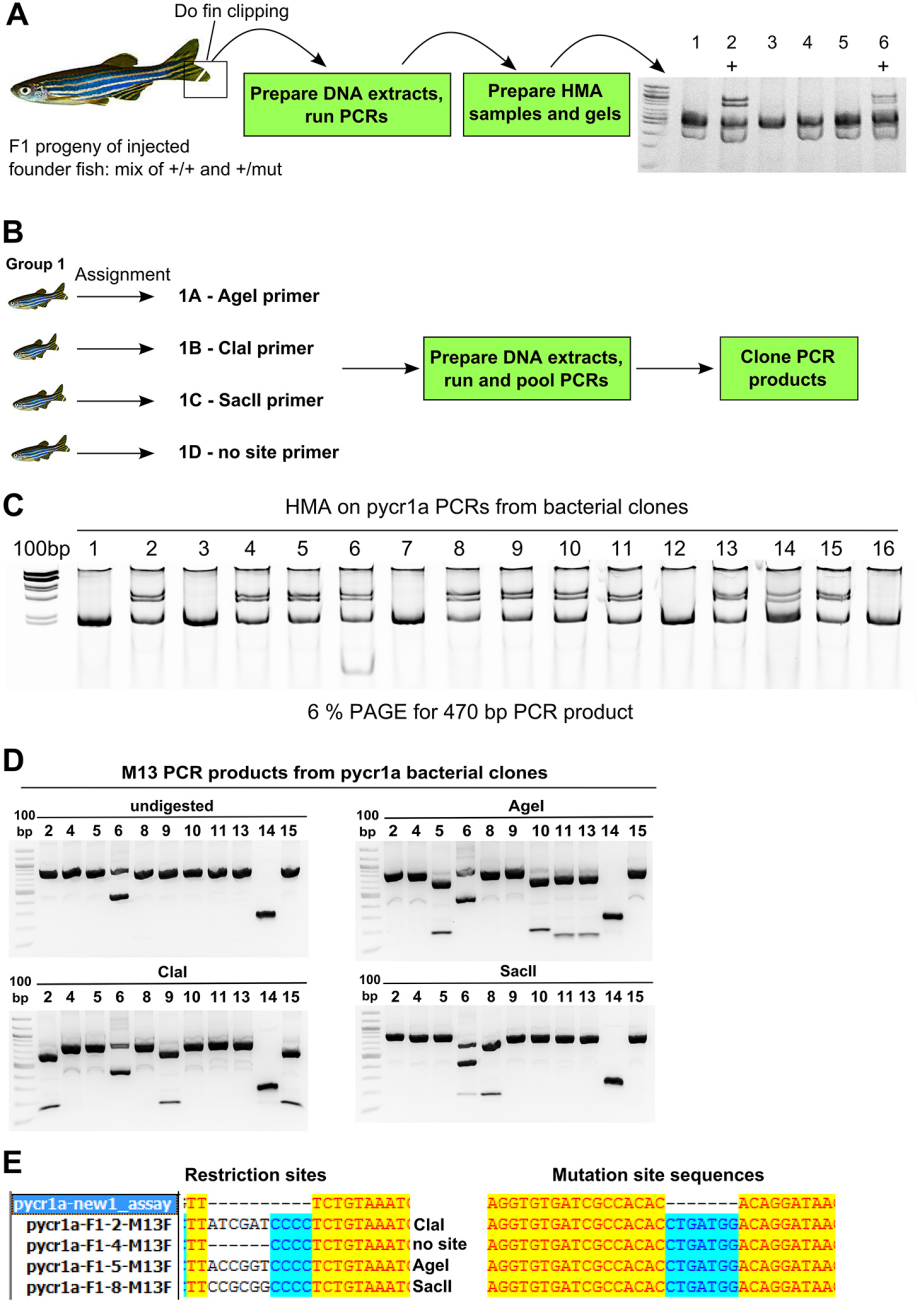
**Strategy for F1 mutation carrier screening and identification of their mutations.** (A) Screening of F1 mutation carriers. Genotyping, mutation screening and sequencing in this strategy are illustrated by data from screening of *pycr1a* mutation carriers. F1 mutation carriers are screened by fin clipping, preparing DNA extracts and running PCRs followed by HMA and the sample results of screening six F1 fish are shown (2 and 6 are positive and marked with ‘+’). (B) Cloning restriction site-tagged PCR products from multiple mutation carriers. Positive mutation carriers are split into groups of four, separated in individual tanks and assigned identifiers (e.g. 1A) and the corresponding forward PCR primers with either *Age*I, *Cla*I or *Sac*II. PCRs with the assigned forward primers and common reverse primer were run on DNA extracts from F1 fish as per assignment, pooled and cloned using a TOPO-cloning procedure. (C) HMA analysis of *pycr1a* bacterial clones. HMA on colony PCR products from bacterial clones mixed with wild-type PCR products is performed and positive clones are identified. (D) Restriction analysis of M13 PCR products. PCR products from positive bacterial clones amplified with M13 primers were digested with enzymes, for which sites were inserted into forward PCR primers, and clones digestible with each of the enzymes are identified. (E) Sequencing analysis of selected *pycr1a* clones. The identified *pycr1a* plasmid clones corresponding to single F1 zebrafish were sequenced and analyzed both at the restriction site position and the mutation site showing complete agreement with previous assays.

To demonstrate a protocol for identifying F1 mutation carrier fish and obtain high-quality sequences of these mutations, we focus on genotyping F1 fish carrying mutations in exon 3 of the *pycr1a* gene, a pyrroline-5-carboxylate reductase ortholog, located on zebrafish chromosome 3 with a gene size of 8 kb and transcript and coding sequence of about 2.5 and 1 kb, respectively. We first found F1 fish carrying mutations in *pycr1a* by performing HMA using a PCR assay for the sgRNA target site (Fig. 1A). The obvious way to sequence these mutations would be to sequence PCR products from mutation carriers, but this strategy is not recommended as it often produces unreliable results owing to sequencing failures or difficulties of resolving normal and mutant sequence chromatograms. In cases when PCR products can be successfully sequenced, software such as PolyPeakParser can help discern the exact mutations from heterozygous PCR product chromatograms (Hill et al., 2014). Some labs with easy and inexpensive access to next-generation sequencing can use this technology to sequence mutations in their positive F1 fish, but in this paper we focus on obtaining plasmids for Sanger sequencing of mutations. After identifying 12 positive F1 *pycr1a* fish, we divided them into three groups with four fish in each, transferred them into individual tanks and assigned a forward primer tagged with a particular restriction enzyme (*Age*I, *Cla*I, *Sac*II or without any site) to each fish in the group (Fig. 1B). Fin clipping and amplification of PCR products with tagged forward primers and a common reverse primer was performed, the PCR products from fish in each group were pooled and TOPO-cloned (Fig. 1B). This approach reduces the number of cloning reactions, plates and samples for sequencing necessary for characterization because it enables researchers to find one bacterial clone corresponding to each F1 carrier fish and thus avoid tedious multiple sequencing submissions. In the next step, PCR product inserts in bacterial clones were amplified using *pycr1a* untagged primers and standard M13 forward (−21) and reverse primers in separate reactions; wild-type *pycr1a* PCR products were also produced. The bacterial colony PCR and wild-type PCR products were then mixed and HMA gels run (see Fig. 1C for an example of results). M13 PCR products from bacterial clones, which test positive in the HMA assay, were digested with *Age*I, *Cla*I and *Sac*II in separate reactions and analyzed on agarose gel electrophoresis together with undigested PCR products (Fig. 1D). This method works because in the pCR2.1 vector the site of M13 primers is separated by about 90-100 bp from the restriction site in the cloned PCR product. We selected clones containing each of the tag restriction sites or no sites at all based on restriction digestions (Fig. 1D) and confirmed these results by sequencing (Fig. 1E). Interestingly, all of the F1 fish we identified in this analysis contained the same seven base-pair (bp) insertion (Fig. 1E) suggesting their origin from the common founder.

### Engineering a vector system for testing frameshifting efficiency of indels by a fluorescent mutation reporter assay

**Fig. 2.**
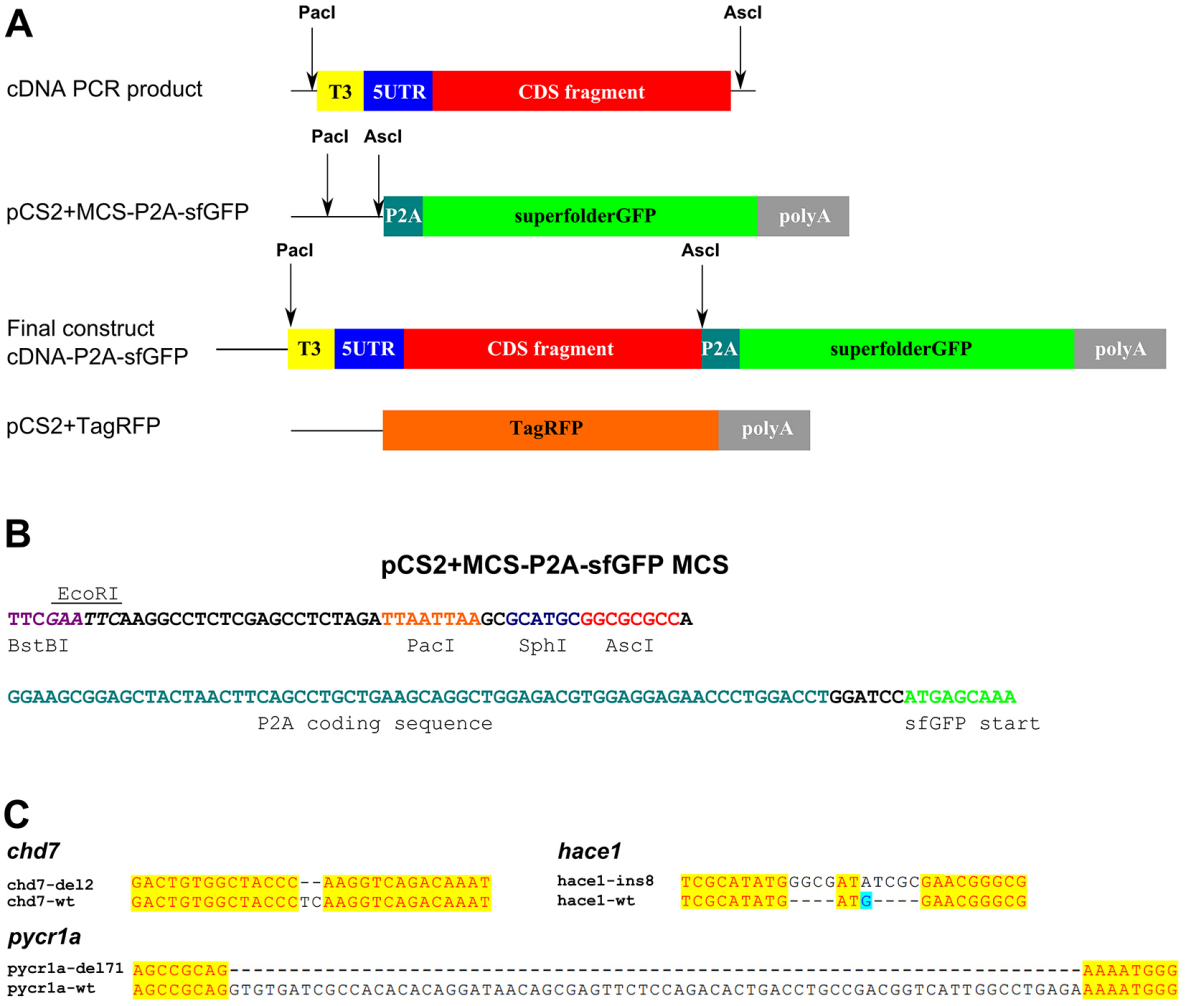
**Cloning strategy for producing mutation reporter constructs using pCS2+MCS-P2A-sfGFP vector.** (A) Mutation reporter vector structure and cloning strategy. PCR products are amplified from cDNA with the primers designed to introduce the T3 polymerase promoter and *Pac*I and *Asc*I sites for cloning and to amplify the 5′ UTR and 200 codons of coding sequence for insertion into pCS2+MCS-P2A-sfGFP vector. (B) The multiple cloning site sequence (MCS) of pCS2+MCS-P2A-sfGFP contains *Bst*BI, *Eco*RI, *Pac*I, *Sph*I and *Asc*I restriction sites followed by the P2A sequence and sfGFP coding sequence. (C) Gene mutations in cloned cDNA fragments. Mutations identified at the level of cDNA in *chd7* (deletion of 2 nt), *hace1* (insertion of 8 nt) and *pycr1a* (deletion of 71 nt) are shown using alignment of mutant sequences to wild-type ones.

The problem with many indel mutations located within the first 100-200 nucleotides of protein-coding genes is that their frameshifting effects can be circumvented by alternative translation initiation. On the other hand, targeting the regions further to the 3′ end of mRNA increases the part of the gene that would be expressed thus increasing the chance of a hypomorphic mutant. One way to circumvent this problem is to induce deletion mutations using several guide RNAs and Cas9, but unless your deletion strategy removes the whole gene, you will still benefit from verifying that the frame of the remaining part of the coding sequence has been shifted. Besides, strategies for gene inactivation using one sgRNA per gene are still fairly common, especially in high-throughput settings. Thus, researchers using zebrafish and other model systems generally need some tools to verify whether the mutations in the protein genes do indeed result in a loss of function due to frameshifts. Antibodies would be one of the best options to achieve this aim, but they are not often available for zebrafish and some other model species. For verification of frameshifting efficiency, we have instead engineered cDNA fusions from wild-type and mutant genes with a superfolder GFP (sfGFP) gene, in which these coding sequences are separated by the P2A sequence used for co-translational cleavage of polypeptides (Kim et al., 2011) in order to ensure that levels of sfGFP expression from such hybrid constructs are independent of the stability of the proteins expressed by the insert cDNA. Translation in the correct reading frame will result in expression of sfGFP from such hybrid transcripts. Because alternative initiation is most likely to occur at the start of mRNAs, a reasonable strategy is to clone the 5′ UTR and about 200-300 codons from the coding sequence of a gene. We applied these principles to engineer a pCS2+MCS-P2A-sfGFP vector for generating mutation reporter constructs, which is based on the popular pCS2+ vector for mRNA expression and contains a multiple cloning site sequence with *Pac*I and *Asc*I rare-cutter restriction enzymes as well as alternative enzymes *Bst*BI, *Eco*RI and *Sph*I, which allow insertion of the cDNA fragments consisting of T3 promoter, 5′ UTR and the coding sequence part to be fused with the P2A-sfGFP coding sequence (Fig. 2A,B). This vector is available from Addgene (vector #74668, deposited by Sergey Prykhozhij) and can be used both for mutation reporters as described in this paper and for expressing complete cDNAs. The reason for the inserts to contain T3 followed by a 5′ UTR is to ensure that the mutation reporter RNA has exactly the same 5′ UTR as the endogenous transcript. To measure the frameshifting efficiency of a particular mutation, one can clone both wild-type and mutant versions of a cDNA and produce corresponding mRNAs by *in vitro* transcription. The final part of the mutation reporter vector system is a pCS2+TagRFP vector, which is used for producing reference TagRFP mRNA for normalizing sfGFP intensity due to injection variability.

### Characterizing and testing mutation reporters on *chd7*, *hace1* and *pycr1a* gene mutations

**Fig. 3.**
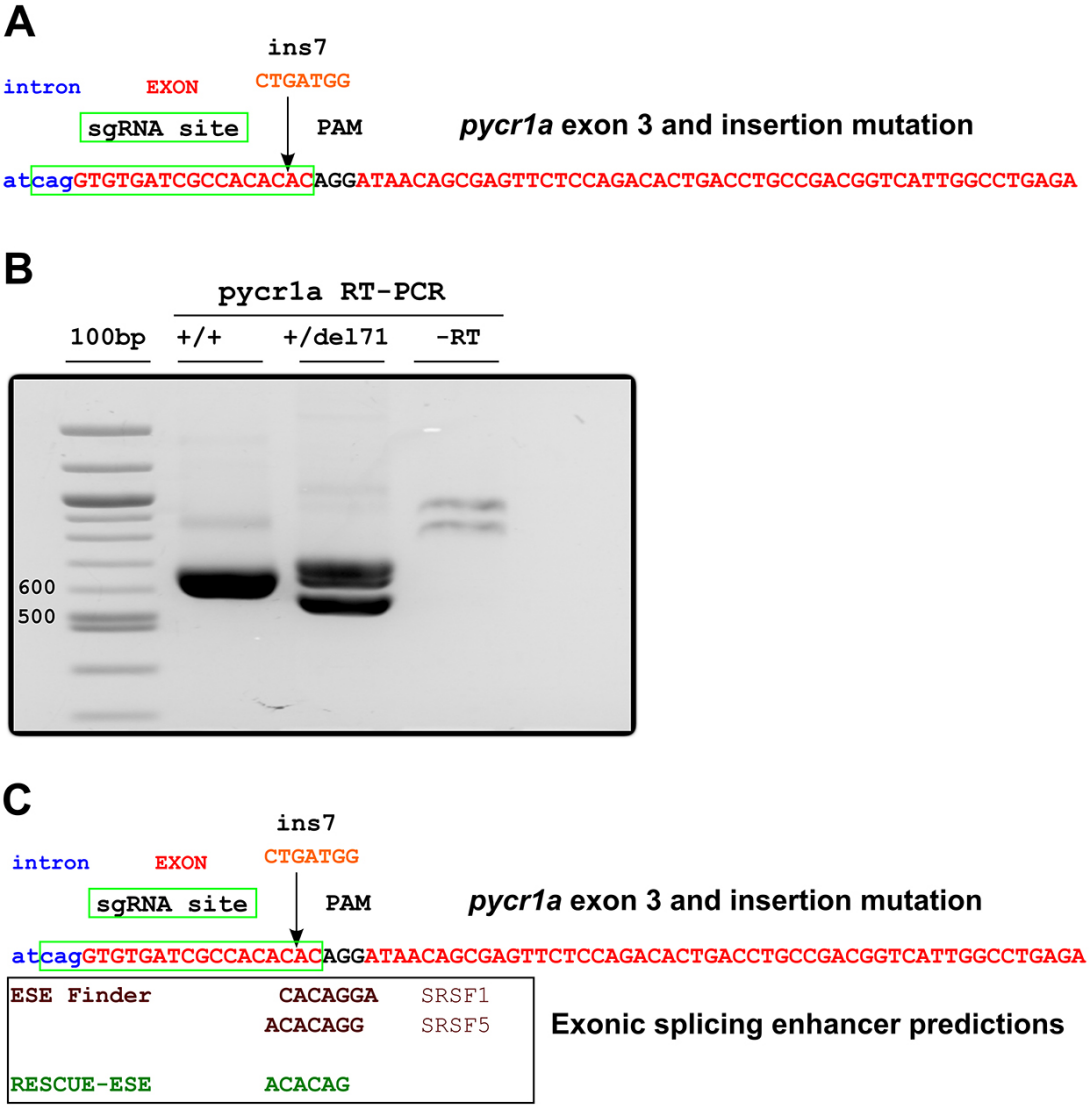
**Mutation in *pycr1a* exon 3 disrupts predicted exonic splicing enhancers.** (A) An insertion of CTGATGG (ins7) was introduced by CRISPR/Cas9 targeting at the sgRNA site 2 bp upstream (indicated by an arrow) from the PAM sequence. sgRNA target site, PAM, intron and exon sequences are indicated. (B) RT-PCR detection of deletion in *pycr1a* cDNA with the skipped exon 3. Amplification of *pycr1a* cDNA fragments for insertion into mutation reporter constructs shows evidence of exon skipping (cDNA fragment with deletion). (C) Exonic splicing enhancers disrupted by ins7 mutation. Exonic splicing enhancer predictions were performed using ESE Finder 3.0 and RESCUE-ESE. For the ESE Finder enhancers, the protein matrix used is indicated and for the RESCUE-ESE, the hexamer overlapping the insertion site is shown.

To demonstrate application of the mutation reporter vectors, we focused on *chd7*, *hace1* and *pycr1a* mutations, for which F1 carriers have been identified using the procedures presented in Fig. 1. The *hace1* gene, encoding HECT domain- and ankyrin-repeat-containing E3 ubiquitin protein ligase 1, is located on chromosome 16 in the zebrafish genome with a gene size of 35 kb and a transcript of 3 kb. This gene has been described to have tumor-suppressor function by antagonizing the production of reactive oxygen species (Daugaard et al., 2013). The *chd7* gene, coding for chromodomain helicase DNA-binding protein 7, a chromatin remodeling factor, is located on chromosome 2 of the zebrafish genome and is a large gene of 55 kb with two main transcripts of 8.6 and 10 kb. In humans, *CHD7* has been identified as the primary causative gene in the development of CHARGE syndrome, a complex disorder of multiple birth defects varying in severity and presentation, with up to 80% of patient cohorts presenting with a heterozygous loss-of-function mutation in the gene (Bergman et al., 2011; Blake and Prasad, 2006; Vissers et al., 2004). All of the mutations in these genes were generated using CRISPR/Cas9-based targeting and are located at sites either overlapping the normal start codon in exon 1 (*hace1*) or 88 and 82 nucleotides (nt) downstream from the start codon in exon 3 of *chd7* and *pycr1a*. Thus, the goal of generating a complete loss of function by such mutations might be obviated by alternative translation start sites if these sites are downstream from the mutation. An 8-bp insertion into the first exon of *hace1* will result in termination after 11 codons if the mRNA is translated from the main start codon, but could lose as few as five codons if the first available alternative site is used. In *chd7* cDNA, deletion of 2-bp (del2) results in a 42-codon ORF from the main start site and the next possible start codon lies in a non-optimal context and overlaps the short mutant ORF by 20 nt. Interestingly, in the case of *pycr1a*, a 7-nt insertion mutation (ins7) identified at the genomic level, at the actual cDNA level, is a deletion of 71 nt (del71) caused by skipping of the targeted exon (Fig. 1E; Fig. 2C; Fig. 3). This represents an instructive example of how inferences from sequencing genomic mutations can be wrong when compared with the actual mutant cDNA. This 71-nt deletion is predicted to result in a frameshift mutant with the stop codon in the main ORF after 31 codons and a potential null allele. However, if one of the next available in-frame start codons was used, this mutation would remove at least 46 amino acids of the Pycr1a reductase domain. In this case, whichever scenario is correct, the function of the protein will likely be disrupted.

In the case of the ins7/del71 *pycr1a* mutation, it is very interesting to study the reasons for exon skipping because such knowledge could be beneficial to either avoid targeting sites promoting exon inclusion and correct splicing or to actively identify and target such sites when the purpose is to artificially induce skipping of particular exons, for example when creating models of certain human mutations that cause exon skipping. One of the common reasons for exon skipping is mutations in exonic splicing enhancers (ESE), which are special sequences in exons bound by splicing factors such as SRSF proteins at the level of primary transcripts that enable or enhance splicing of these exons (Cartegni et al., 2002; Desmet and Beroud, 2012). Many examples of exonic mutations, including silent and missense mutations, have been identified in exons of patients suffering from different genetic diseases (Cartegni and Krainer, 2002; Desmet and Beroud, 2012; Gonçalves et al., 2009; Hahn et al., 2009; Zatkova et al., 2004). To examine whether disruption of ESEs underlies this instance of exon skipping, we ran RESCUE-ESE (Fairbrother et al., 2002, 2004) and ESE Finder (Cartegni et al., 2003) programs on the sequence of the *pycr1a* exon 3. Both of these programs identified ESE sequences that overlapped the sgRNA target site protospacer adjacent motif (PAM) sequence and the site where ins7 occurred (Fig. 3). Importantly, the ESEs identified by ESE Finder were top-scoring sites in the exon sequence with several others that were either identical or overlapping the two shown in Fig. 3. Thus, the results of these bioinformatics analyses strongly suggest that disruption of ESEs by the ins7 mutation causes exon skipping in *pycr1a*. This idea is also supported by observations indicating the crucial importance of ESEs for splicing of relatively short exons, such as exon 3 of *pycr1a*, and those located closely (14-15 nucleotides in this case) to the acceptor splice site (Cáceres and Hurst, 2013).

**Fig. 4.**
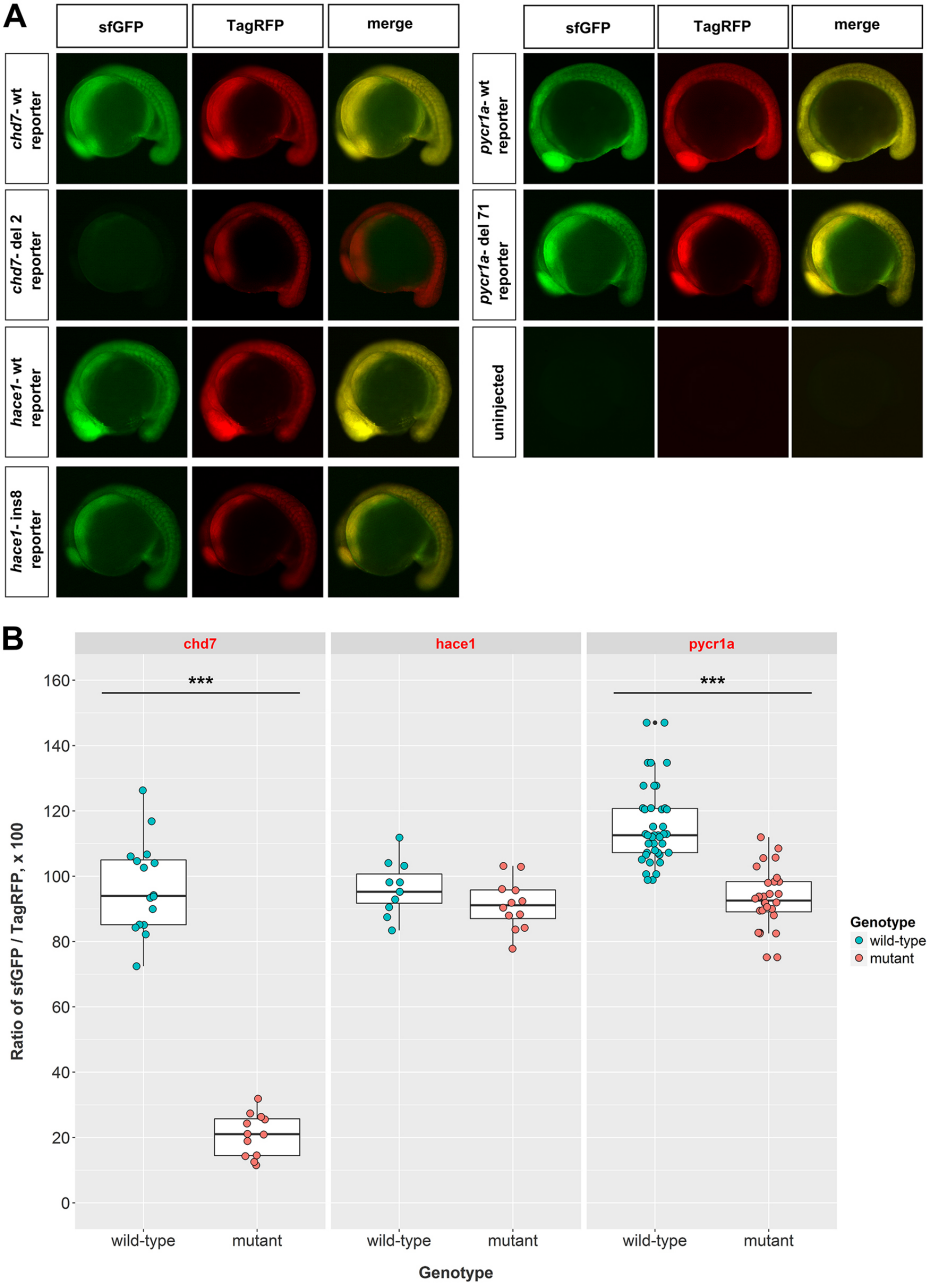
**Expression of mutation reporters for *chd7*, *hace1* and *pycr1a* mutations quantifies their frameshift efficiency.** (A) Representative images of 16-18 hpf (∼16-somite stage) zebrafish embryos injected with the indicated mutation reporter mRNA from constructs made as described in Fig. 3 or uninjected. Fluorescence images of superfolder GFP (sfGFP) and TagRFP and merged images are shown. (B) Areas in the somite regions of these embryos were quantified by measuring fluorescence intensities of both sfGFP and TagRFP, adjusted for background in the corresponding channel, and the ratio of these intensities was calculated to account for injection variability and used for plotting the data and statistical analysis. For *chd7* mutation reporters, the difference between wild-type (*n*=15) and 2-bp deletion mutant (*n*=12) mutation reporters was large and very significant (*P*=2.034e−15). Embryos injected with *hace1* wild-type (*n*=10) and 8-bp insertion mutant (*n*=12) cDNA fragments did not show any significant difference. The *pycr1a* wild-type (*n*=41) and 71-bp deletion mutant (*n*=28) reporters had a small but significant difference (*P*=7.776e−13). ***P<0.001. The injections were performed twice on different days with very similar results and embryos from one of the injections were subjected to imaging and statistical analysis of fluorescent protein intensities ratios. Student's two-tailed *t*-test was performed for each experiment.

To provide proof-of-principle evidence that frameshift efficiency of mutations can be measured directly in zebrafish embryos, we cloned wild-type and mutant 200-codon cDNA fragments of *chd7*, *hace1* and *pycr1a* using the previously described approach (Fig. 2A). The *in vitro* synthesized mRNAs from the vectors containing cloned cDNA fragments in-frame with sfGFP were measured, verified for integrity on agarose gels and co-injected with TagRFP to achieve nearly equal expression levels of both fluorescent proteins from the wild-type mRNAs. Imaging injected embryos at 16-18 hours post-fertilization (hpf) showed a striking reduction of mutation reporter expression only in the case of *chd7* del2 mutant (Fig. 4A). However, to make the results quantitative, we measured equivalent regions of somites in all of the embryos in both fluorescent channels and calculated ratios of sfGFP to TagRFP expression to normalize for variable injection volumes. As expected, this analysis revealed a large and significant decrease in sfGFP mutation reporter level for the *chd7* del2 mutation (Fig. 4B). There was also a significant, albeit relatively weak difference, between wild-type and mutant mutation reporter levels for *pycr1a* (Fig. 4B). For *hace1*, no difference between wild-type and mutant mutation reporters was observed (Fig. 4B) suggesting potent alternative translation start sites or re-initiation after translation terminates due to the frameshift. To test whether a more downstream mutation in the *hace1* gene would result in effective frameshifting, we artificially introduced a 4-bp deletion after the first 106 codons. Such a mutation could, in principle, be created by CRISPR/Cas9 targeting at that site or close to it. The mutation reporter experiments comparing the wild-type and 4-bp deletion *hace1* reporters showed that this deletion induced very efficient frameshifting of about 90% (Fig. S1A,B). Overall, this analysis demonstrates the results for a workflow aimed at quantifying frameshift efficiency in mutant mRNAs by their comparison to wild-type mRNAs using mutation reporter constructs.

**Fig. 5.**
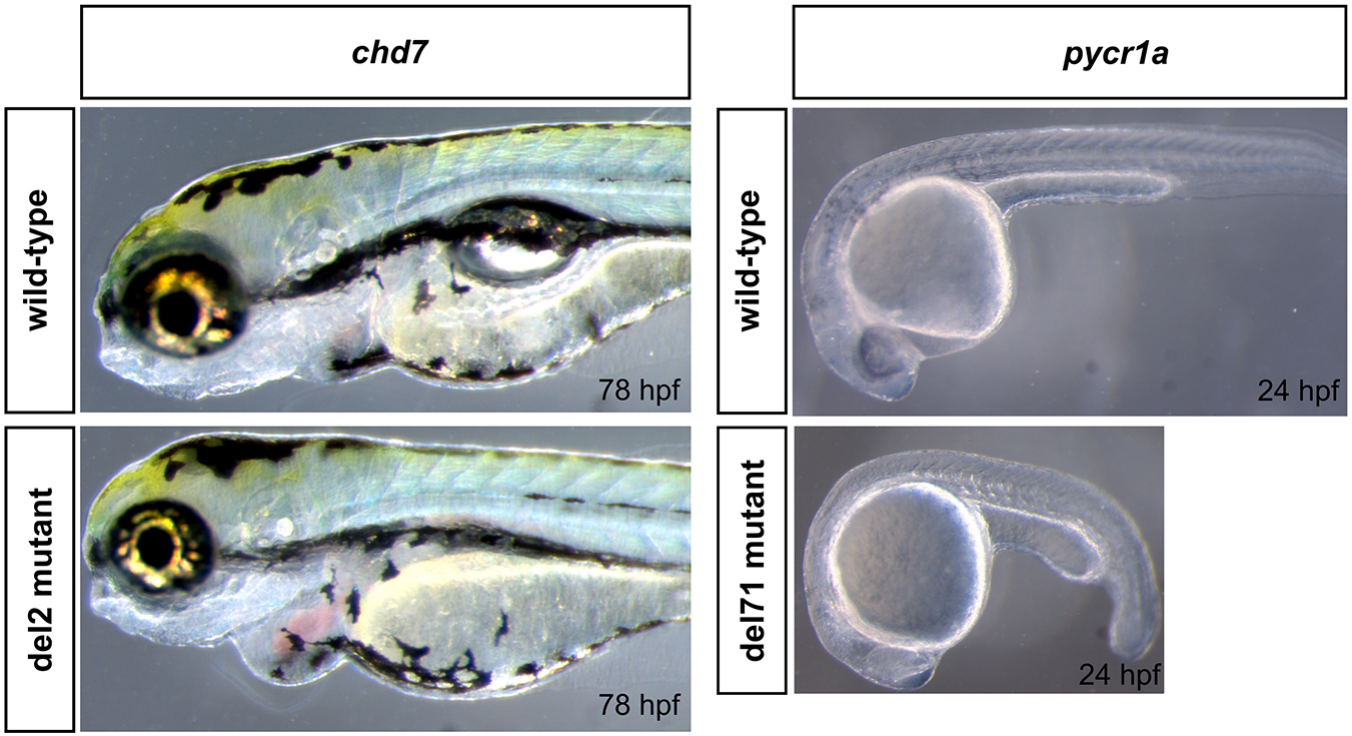
**Phenotypes of *chd7* and *pycr1a* mutants.** The phenotype of *chd7* del2 homozygous mutants was most clearly observed after 72 hpf and included smaller eyes, enlarged heart with edema and failure to inflate the swimbladder; images show fish at the 78 hpf stage as an example. For *pycr1a* del71 mutants, the observed abnormality was a pronounced developmental delay at 24 hpf, as shown in the image, that persists up to about 48 hpf and disappears afterwards.

We also analyzed the phenotypes of the zebrafish mutants presented in this study to assess the functional relevance of mutation reporter assays. The incrosses of *chd7*+/del2 fish consistently yielded about 25% of embryos, which after 72 hpf showed enlarged hearts with edema, smaller eyes than in wild type and failure to inflate swimbladder (Fig. 5). This result is strongly consistent with the mutation reporter assays for the *chd7* del2 mutation. For the *pycr1a* +/del71 incrosses, we consistently observed that about 25% of embryos had a pronounced delay in development (Fig. 5), which, however, gradually disappeared after 48 hpf. An observation of a mutant phenotype resulting from *pycr1a* del71 mutation thus indicates that the predicted negative effect on Pycr1a protein is functionally significant but this might be later compensated for by another paralog such as *pycr1b*. Finally, we did not observe any significant phenotypes in embryos from *hace1*+/ins8, which is potentially consistent with the mutation reporter results, but can also be explained by the subtle nature of the phenotype due to translation of the mutant protein from an alternative start site.

## DISCUSSION

Methods for applying CRISPR/Cas9 technology for generating mutants in zebrafish are available (reviewed by Gonzales and Yeh, 2014; Varshney et al., 2015b). To establish that a particular mutation represents a null allele, it is necessary to isolate the animal and sequence and analyze the mutation to determine whether the ORF of the gene is affected. However, screening heterozygous mutation carriers and determining their mutations can still be laborious and time-consuming. We have built upon multiple currently available approaches to provide a comprehensive workflow for screening F1 mutation carriers, cloning and sequencing their mutations followed by assays of their frameshifting efficiency.

Detection of mutations in heterozygote zebrafish using HMA (Chen et al., 2012; Ota et al., 2013, 2014) is one of the simplest and most robust methods for this purpose. To enable wider adoption of this technique, we have provided a highly detailed protocol of HMA for PCR products of different sizes (see supplementary information). Furthermore, in this workflow, we have applied multiplexing and barcoding of samples with restriction enzyme sites to match single fish to bacterial clones containing plasmids with mutant PCR product inserts in order to minimize the number of sequencing reactions required to identify mutations in F1 mutation carriers. Sequencing plasmids with cloned PCR products is a more reliable but more time-consuming approach in this context than sequencing PCR products from heterozygous samples and interpreting them (Hill et al., 2014; Talbot and Amacher, 2014). Thus, it is advisable to initially sequence PCR amplicons from heterozygous F1 fish and then apply PolyPeakParser software to the results. If this initial attempt fails or is not fully conclusive, the strategy of cloning and screening of mutant PCR product plasmid clones should be applied. Once the mutations are identified in each F1 zebrafish being screened, they can be prioritized for phenotypic screening and establishment of F2 mutation-carrying families.

In theory, it is straightforward to predict which mutations can cause a shift in the currently accepted normal reading frame, thus theoretically leading to an early termination of translation and possible mRNA decay, which are hallmarks of null mutations. However, more than half of genes expressed in mouse embryonic stem cells have alternative translation start sites (Ingolia et al., 2011) and this could also be the case in zebrafish. The problem is that these alternative translation start sites are still poorly annotated, so when generating frameshift mutations by using CRISPR/Cas9, it might not be possible to predict the targeting site closest to the start codon that cannot be bypassed by alternative translation sites. Thus, simple inferences based on sequencing of mutations might be unreliable. On the other hand, targeting too far upstream from the start codon could result in hypomorphic mutants or expression of truncated protein products. Another potential concern is that targeting early exons might be insufficient for generating the null mutation because of transcription from alternative promoters. Although the information on alternative promoters is limited for zebrafish (Adriao et al., 2016; Chen et al., 2010; Cotter et al., 2013), it should be utilized for mutation-targeting strategies when available. Deletion mutants generated by targeting genes using multiple sgRNAs (Ota et al., 2014; Xiao et al., 2013) can bypass the problems of alternative translation initiation and promoters, for example if one targets a highly conserved and/or important domain in the middle of a gene. The efficiency of deletion strategies varies widely in our own experience and can reach high frequencies when sites for highly active sgRNAs are positioned closely. Despite the potential advantages of CRISPR/Cas9-based deletion strategies, frameshift mutants at single targeting sites are still common and might even be preferable for certain high-throughput applications. Another useful strategy is to engineer two indel mutations in different parts of the gene and verify that the phenotypes are identical. We envisage that our current mutation reporter approach will be useful for measuring frameshift efficiencies at both mutated sites. Such measurements would benefit the researchers if the different mutations show different phenotypes or if the phenotypes are not obvious with the available methods of assessment. The different phenotypes could be due to off-target effects or due to problems with one of the mutants, such as alternative translation initiation or alterations of splicing.

In most analyses of CRISPR/Cas9-induced mutations, genomic DNA is assayed, but such analyses do not take into account possible effects of mutations on splicing and alternative splicing patterns. We focused on cDNA to analyze several mutations and clone them into reporter constructs. For a mutation in the *pycr1a* gene, we observed skipping of a complete exon and performed bioinformatics analyses of the mutated sequence, which led to the finding that the mutation disrupted predicted ESEs. To our knowledge, this is the first reported observation of exon skipping by CRISPR/Cas9-induced mutations in ESEs, although this phenomenon has been documented for many human mutations (Desmet and Beroud, 2012). A related observation of exon skipping due to large gene cassette insertions has been described recently (Uddin et al., 2015), although in that case exon skipping was a result of the large size of the newly engineered exon and not due to ESE disruption. Given high-throughput studies of mutations generated by CRISPR/Cas9 techniques such as the CRISPRscan study (Moreno-Mateos et al., 2015), it is surprising that exon skipping was not detected frequently; this might simply reflect the fact that most mutation detection analyses have been carried out on genomic DNA. Our finding of ESEs and their disruption by the *pycr1a* mutation suggests that locating these regions in exons being targeted by genomic engineering approaches might be useful for avoiding exon skipping or, alternatively, for actively inducing it. Software tools for CRISPR/Cas9 sgRNA design could even be designed to include ESE predictions.

To assess which mutations are effective frameshifters, we generated fluorescent reporter plasmid constructs containing 5′ UTR, cDNA fragments, a P2A co-translational cleavage sequence and sfGFP. The size of the cDNA fragments to test in this reporter is an important variable, which for our experiments we set at 200 codons because most alternative start sites have been predicted or found in the first 100 codons (Bazykin and Kochetov, 2011; Gao et al., 2015). However, the vector we used for cloning does not place any restriction on the size of mutation reporters. As a proof of principle, we applied this reporter strategy to three genes, for each of which a wild-type and a mutant cDNA version was cloned. Injection of mRNAs of mutation reporter constructs showed effective frameshift only for a mutation in the *chd7* gene but not for *pycr1a* or *hace1*. This could not have been predicted from the basic knowledge of the structure of these genes and suggests the existence of strong alternative start sites in the other two genes. Moreover, these results are qualitatively different from RT-PCR, gel analysis and sequencing as none of these methods predicted which of the mutations analyzed in this study would disrupt ORFs. The phenotypic analyses of mutants showed conclusively that mutations in *chd7* and *pycr1a* genes produce significant and robust phenotypes, further reinforcing the validity of the results of mutation reporters and mutant cDNA analyses, respectively. When one identifies that a mutation does not effectively shift the reading frame, it is still possible to infer what could be another available initiation codon. It is then possible to infer whether the resulting protein truncation is likely to be detrimental. We have done this in the context of *pycr1a* and this analysis strongly suggested that the Pycr1a reductase domain is disrupted by the mutation. An approach similar to mutation reporters for verifying frameshift efficiency of mutations has previously been applied to *pak4* gene mutations, for which Pak4-EGFP fusion with wild-type cDNA showed strong fluorescence when fusion gene mRNA was injected into zebrafish, whereas the indel version of *pak4* cDNA lacked EGFP fluorescence (Law and Sargent, 2014). While our manuscript was under review, Sive and colleagues published an *in vitro* translation approach for protein knockout validation in zebrafish (Carter et al., 2016). This approach is conceptually similar to ours in that it also involves cloning cDNAs of wild-type and mutant genes, but differs from our protocol as the read-out is produced by analyzing *in vitro* translations for the level of proteins with incorporated biotin-labeled lysine residues using streptavidin-based western blotting. This method has the advantage of visualizing alternative initiation or truncation events without the use of tagging strategies. We have attempted to apply this *in vitro* translation procedure to the mutants described in this study, but we were unsuccessful (S.V.P., unpublished observations). Thus, we propose that our mutation fluorescent reporter strategy not only has the benefit of being an *in vivo* readout of protein translation, but might also be more technically achievable than an *in vitro* translation procedure for mutant validation.

Antibody staining is a more traditional method for frameshift mutation analysis. From a practical perspective, incrosses of F1 heterozygotes will produce homozygotes or compound heterozygotes with two different mutations. For zebrafish proteins with good antibodies, the putative mutant clutches can be used for western blotting if phenotypic selection is possible or for immunofluorescence if phenotypes are not apparent. However, only a minority of zebrafish proteins have high-quality and proven antibodies available. Mutation reporters can thus be useful for testing which mutations in protein-coding genes are null even when antibodies are not available or cannot be applied. As a dearth of good antibodies is a challenge for many novel model species, applying the overall workflow and mutation reporters described in this article or fluorescent protein fusions to screen and identify mutations and assay their frameshift effectiveness of mutations could serve as a preferred and more universal strategy to complement phenotypic analysis of the same mutations. As with any tool, mutation reporters have limitations, such as the fact that for effective frameshifting mutations the reporters will not help identify exactly where translation terminated. More importantly, for cases in which alternative translation sites are active, the current version of the vector will not identify the sizes of alternative protein products. This latter problem can be addressed using reporters based on protein fusions or epitope tags. For example, when cloning cDNA fragments for testing mutation frameshift efficiency into our vectors or fluorescent protein fusion vectors, one can include a C-terminal epitope tag and then perform western blotting of wild-type and mutant reporter mRNAs to determine the sizes of alternatively initiated proteins. Another possibility is to establish an epitope-tagged or protein fusion animal model and then directly test frameshift efficiency of mutations directly *in vivo*. The latter strategy is much more time-consuming, but may be worth the effort if a particular lab requires both the epitope-tagged and mutant versions of a particular gene.

In conclusion, we think that the approaches presented in this article will facilitate the fundamental analysis of gene functions and the establishment of disease models in zebrafish and other model systems.

## MATERIALS AND METHODS

### Animal care and husbandry

Zebrafish housing, breeding conditions, and developmental staging of larvae were performed according to Westerfield (2007). Use of zebrafish in this study was approved by the Dalhousie University Committee on Laboratory Animals (Protocols 15-134; 15-123). All zebrafish embryos were maintained in E3 embryo medium (5 mM NaCl, 0.17 mM KCl, 0.33 mM CaCl_2_, 0.33 mM MgSO_4_) in 10 cm Petri dishes at 28°C. Mutant fish were generated in the following backgrounds: *pycr1a* (Tubingen, TUB), *hace1* (AB) and *chd7* (TUB). Casper pigment mutant fish were used for performing the fluorescent reporter assays (White et al., 2008).

### Heteroduplex mobility assay for identification of F1 mutation carriers

Adult fish were anesthetized in fish water containing Tricaine (0.02%), then pieces of their tail fins were collected into 30 µl of 50 mM NaOH. The fin clip samples were boiled for 10 min at 95°C in a PCR machine, vortexed, cooled on ice and neutralized by addition of 3 µl of 1 M Tris-HCl pH 8.0. We ran PCR using Taq DNA polymerase (ABM, G009) in 20 µl reaction volume with the buffer, dNTP (0.2 mM), 0.25 µM of each forward and reverse primers, 2 µl of sample and 0.2 µl of Taq. Amplification was performed for 36 cycles with annealing temperature of 55°C and extension time of 30 s. PCR reactions were then denatured and slowly cooled according to this procedure: 95°C for 5 min, 85°C for 2 min followed by cooling to 25°C at a rate of 0.1°C/s. Before loading on the gel, 2 µl of SYBR Green I (Sigma-Aldrich, S9430) diluted to 100× from 10,000× stock in DMSO and 2 µl of 6× Gel Loading Dye, Purple (New England Biolabs, B7024S) were added to each sample.

Polyacrylamide gels for DNA analysis were prepared according to McGookin (1988) with the 20× Gel Running Buffer containing 1 M Tris, 1 M boric acid, 200 mM EDTA, pH 8.3. Gel volume sufficient for two gel cassettes (15 ml) at 6% contained 0.75 ml of 20× Gel Running Buffer, 11.13 ml of water, 3 ml of 30% acrylamide/bis-acrylamide (29:1) (Bio-Rad, 1610156), 105 µl of 10% ammonium persulfate (Sigma-Aldrich, A3678), 15 µl of TEMED (Sigma-Aldrich, T9281). Gels were assembled and run using Mighty Small II Deluxe Mini Vertical Electrophoresis Unit (Hoefer, SE260). Preparation of other gel percentages for different sizes of DNA is described in the supplementary information. The gels were run for 15-30 min at 60 V without the samples, which were then loaded and run further for 1 h.

### Cloning PCR products from multiple F1 mutation carriers and screening

Pooling individual samples is a well-established strategy when creating sequencing libraries and for other purposes. As there is a need to barcode individual samples, the number of samples pooled together or a degree of multiplexing has to be defined. For cloning *pycr1a* mutations from F1 mutation carriers, the degree of multiplexing was four. Once these fish were divided into groups, each fish was assigned a certain primer and a corresponding restriction enzyme. PCRs with forward primers containing *Age*I, *Cla*I or *Sac*II sites or no site were run with samples from these fish (Fig. 1A,B). The resulting PCRs from the same group of fish were checked on the gel, mixed, purified using QIAquick PCR purification kit (QIAGEN, 28104) and TOPO-cloned into pCR2.1-TOPO (Thermo Fisher Scientific, 450641). After transformation, colony PCR was performed on 16 bacterial colonies from a plate for a particular group of F1 mutation carriers. Each colony was resuspended in 100 µl of sterile water, then 10 µl of this suspension was transferred into a separate tube, heated at 95°C for 5 min and 10 µl of Taq master mix were added and the PCR was run. In addition, 100 µl PCR was run using wild-type genomic DNA as template. For all reactions at this step, non-tagged *pycr1a* primers were used. The colony PCR reactions were verified by agarose gel electrophoresis. HMA samples from these PCR reactions were prepared by mixing in the new tubes 5 µl of wild-type PCR reaction with the same volume of colony PCR being identified. Colony PCR mixes were re-hybridized and prepared as described in the HMA protocol. After identifying which bacterial clones contain mutant PCR products, we checked which fish these clones correspond to. PCR products from the putative mutant clones were amplified using M13 primers, for which sites are present in many common cloning vectors and which are offset from the inserts by 90-100 bp in pCR2.1. An aliquot (5 µl) from each PCR reaction was digested with *Age*I, *Cla*I and *Sac*II in 20 µl reactions for 2 h at 37°C. The digestions were analyzed on a 2% agarose gel by running agarose gel electrophoresis at 90 V for 1 h. Mini-prep cultures from identified colonies were grown, and plasmids purified and submitted for sequencing.

### Cloning of pCS2+MCS-P2A-sfGFP and pCS2+TagRFP

We created pCS2+MCS-P2A-sfGFP for generating mutation reporter plasmids for any mutant protein-coding gene through several steps. Primers attB1_*Bst*BI-*Not*I-P2A_for and attB2_*Bam*HI-*Eco*RI-P2A_rev were used in an overlap-extension PCR to generate a PCR product, which was recombined with pDONR221 using BP Clonase II (Thermo Fisher Scientific, 11789-020) to produce pME-*Bst*BI-*Not*I-P2A-*Bam*HI-*Eco*RI. Next, superfolderGFP (sfGFP) was amplified using *Bam*HI-sfGFP_for and *Eco*RI-sfGFP_rev and inserted into the pME vector using standard cloning procedures. P2A-sfGFP fragment was amplified from pME-*Bst*BI-*Not*I-P2A-sfGFP using *Xba*I-*Pac*I-*Sph*I-*Asc*I_P2A-sfGFP_for and *Xba*I_P2A-sfGFP_rev to introduce a multiple cloning site (MCS) and *Xba*I sites for insertion into pCS2+. The resulting *Xba*I-MCS-P2A-sfGFP-*Xba*I PCR product was digested with *Xba*I and inserted into pCS2+ linearized with *Xba*I and dephosphorylated with CIP (New England Biolabs, M0290S). We also generated pCS2+TagRFP to produce reference TagRFP mRNA for injections by amplifying TagRFP insert using *Bam*HI-TagRFP_for and *Eco*RI-TagRFP_rev and inserting it into pCS2+.

### Cloning of mutation reporter plasmids

We bred *hace1*, *pycr1a* and *chd7* F1 mutations carriers to wild-type fish, collected embryos at 48 hpf and extracted RNA using RNeasy Mini Kit (QIAGEN, 74104). cDNA was made by mixing 10 µl of total RNA with 4 µl of 2.5 mM dNTP and 2 µl of 100 µM oligo-dT(18) (Integrated DNA Technologies), heated at 70°C for 10 min and cooled on ice. Alternatively, one can use 2 µl of 5 µM a gene-specific reverse primer instead of oligo-dT. The reaction was completed by adding 2 µl of M-MuLV buffer, 0.25 µl of RNAsin (New England Biolabs, M0314S), 0.25 µl of M-MuLV reverse transcriptase (New England Biolabs, M0253S) and 1.6 µl of water. cDNA synthesis was performed at 42°C for 1 h and at 90°C for 10 min. The primers were designed to amplify a cDNA fragment containing a 5′ UTR and the first 200 codons from the coding sequence. The forward primer contained the T3 phage RNA polymerase promoter as well as a *Pac*I restriction site, and the *Asc*I site was present in the reverse primer (Table S1). cDNA fragments of *hace1*, *pycr1a* and *chd7* genes with or without mutations were amplified from corresponding cDNA samples using Q5 polymerase (New England Biolabs, M0491S) according to the standard protocol with an annealing temperature of 61°C. The resulting PCR products were run on agarose gel and extracted using QIAquick Gel Extraction kit (QIAGEN, 28704), digested with *Pac*I and *Asc*I (New England Biolabs, R0547S and R0558S), purified and inserted into pCS2+MCS-P2A-sfGFP using standard cloning methods. At least four clones were sequenced to find at least one wild-type and one mutant variant from each cloning. Site-directed mutagenesis of the *hace1* wild-type mutation reporter construct was performed by amplifying the whole plasmid using Phusion polymerase (New England Biolabs, M0530S) from 1 ng of plasmid DNA using hace1-2nd-mut-SDM_for and hace1-2nd-mut-SDM_rev primers (Table S1). The PCR product was then digested with *Dpn*I enzyme (New England Biolabs, R0176S) and gel-extracted. Combined T4 PNK (Thermo Fisher Scientific, EK0031) and T4 ligase (New England Biolabs, M0202S) reaction was assembled containing 1 µl of 10× T4 ligase buffer, 1 µl of each enzyme and 7 µl of the PCR product, incubated for 1 hour and transformed into OneShot TOP10 competent cells (Thermo Fisher Scientific, C404003).

### Production of RNA from mutation reporter vectors and zebrafish injections

The mutation reporter vectors and pCS2+TagRFP were linearized by digestion with *Not*I, extracted with UltraPure Phenol:Chloroform:Isoamyl Alcohol (25:24:1) (Thermo Fisher Scientific, 15593-031) in Phase Lock Light 1.5 ml tubes (5 PRIME, 2302800) and precipitated by adding 1/10th volume of 3 M sodium acetate pH 5.5, two volumes of ethanol and 0.5 µl of glycogen (Sigma-Aldrich, G1767), chilling at −20°C and spinning at the maximum speed for 15 min. mRNA synthesis was performed using mMESSAGE mMACHINE T3 Transcription Kit (Thermo Fisher Scientific, AM1348) in 10 µl reactions and purified by Phenol:Chloroform:Isoamyl Alcohol extraction according to the manufacturer's instructions. All mRNAs were verified for quantity and integrity by measuring their concentrations and visualizing them by agarose gel electrophoresis. The mutation reporter mRNAs encoding wild-type or mutant cDNA and sfGFP separated by P2A cleavage signal were individually mixed with TagRFP mRNA to the final concentrations of 100 and 50 ng/µl, respectively. These mixes were injected into one-cell stage zebrafish eggs by standard microinjection procedures.

### Imaging and quantification of mutation reporter fluorescent intensities

Somitogenesis-stage embryos (16-18 hpf) were embedded into 1% low-melting-point agarose (Sigma-Aldrich, A9414) in imaging glass-bottom dishes and imaged using Zeiss Axiovision fluorescent microscope for green and red fluorescence. Both injected and uninjected embryos were imaged in both channels. The values for green and red fluorescence were calculated by importing images into Fiji software (Schindelin et al., 2012), splitting their channels and then merging green and red channels into a single stack using Stacks/Tools/Interleave command followed by analyses of regions of interest (ROI) in the somite regions using ROI Manager plugin. The average values from ROI measurements were further normalized by subtracting background from the same channels and images. The resulting values for green and red channels were used to calculate green/red ratios, which correspond to fully normalized values of mutation reporter construct expression. These ratio values were used for statistical analyses and data plotting using the R programming environment.

### Bioinformatics analysis of exonic splicing enhancers

We used RESCUE-ESE (http://genes.mit.edu/burgelab/rescue-ese/) (Yeo et al., 2004) and ESE Finder 3.0 (http://rulai.cshl.edu/cgi-bin/tools/ESE3/esefinder.cgi?process=home) (Cartegni and Krainer, 2002; Smith et al., 2006) for predictions of exonic splicing enhancer in *pycr1a* exon 3 sequence. For the ESE Finder run, we chose to focus on the best hits for each of the SRSF splicing factors to simplify the output. The sequences of the identified ESEs were copied from the output HTML pages and used for Fig. 3.
